# *De novo* assembly and analysis of the transcriptome of *Rumex patientia* L. during cold stress

**DOI:** 10.1371/journal.pone.0186470

**Published:** 2017-10-12

**Authors:** Jianxin Liu, Yongqing Xu, Liguo Zhang, Wei Li, Zhenxue Cai, Fei Li, Mu Peng, Fenglan Li, Baozhong Hu

**Affiliations:** 1 College of Life Science, Northeast Agricultural University, Harbin, China; 2 Heilongjiang Academy of Agricultural Sciences, Harbin, China; 3 Alkali Soil Natural Environmental Science Center, Northeast Forestry University, Harbin, China; 4 Harbin university, Harbin, China; Youngstown State University, UNITED STATES

## Abstract

**Background:**

*Rumex patientia* L. is consumed as a green vegetable in several parts of the world, and can withstand extremely low temperatures (-35°C). However, little or no available genomic data for this species has been reported to date. Here, we used Illumina Hiseq technology for transcriptome assembly in *R*. *patientia* under normal and cold conditions to evaluate how it responds to cold stress.

**Results:**

After an in-depth RNA-Seq analysis, 115,589 unigenes were produced from the assembled transcripts. Based on similarity search analysis with seven databases, we obtained and annotated 60,157 assembled unigenes to at least one database. In total, 1,179 unigenes that were identified as differentially expressed genes (DEGs), including up-regulated (925) and down-regulated ones (254), were successfully assigned GO annotations and classified into three major metabolic pathways. Ribosome, carbon metabolism, oxidative phosphorylation and biosynthesis of amino acids were the most highly enriched pathways according to KEGG analysis. Overall, 66 up-regulated genes were identified as putatively involved in the response to cold stress, including members of MYB, AP2/ERF, CBF, Znf, bZIP, NAC and COR families.

**Conclusion:**

To our knowledge, this investigation was the first to provide a cold-responsive (COR) transcriptome assembly in *R*. *patientia*. A large number of potential COR genes were identified, suggesting that this species is suitable for cultivation in northern China. In summary, these data provide valuable information for future research and genomic studies in *R*. *patientia*.

## Introduction

Temperature substantially influences cytomembrane fluidity and membrane lipid composition, which in turn affect protein folding and gene expression [1]. Temperate plants are tolerant of chilling temperatures (0–15°C) but are usually intolerant of freezing temperatures (< 0°C) [2]. In response to low temperature, the transcriptome and metabolism in plants are strongly altered via regulation of the expression of related genes. After exposure to low temperature, the transcriptome of the model plant *Arabidopsis thaliana* shows massive changes that culminate in a considerable increase in cold-responsive (COR) genes expression, from 4% to 20% [3,4]. In alfalfa (*Medicago sativa*), a decrease in membrane fluidity rapidly induces expression of numerous downstream COR genes, completing the process of the response to cold conditions [5]. Gene mutations and chemical agents can restore cell membrane-induced cold acclimatization-specific gene expression at high temperatures [6,7]. In addition, as a second messenger, the Ca^2+^ signaling pathway is central to the early response to cold stress [8], and many reports have demonstrated that Ca^2+^ activity is essential for cold-shock gene expression. When calcium ion-chelating agents or materials that block calcium channels are introduced to cells, COR gene expression decreases, and chilling stress is simultaneously mitigated. Additionally, an artificial increase in the cytoplasm Ca^2+^ concentration can induce cold stress-specific gene expression, even at warm temperatures [9].

Cold stress induces notable changes in C-repeat binding factors (CBFs) and dehydration-responsive element-binding protein 1 (DREB1), which specifically bind to *cis*-elements in the promoters of COR genes and activate their expression [10,11]. Many genes are regulated by CBFs, which mainly participate in mechanisms that protect cells, including the inositol phospholipid pathway, transcription, reactive oxygen species detoxification, membrane transport, and hormone metabolism. In addition to *Arabidopsis*, COR homologs have been cloned from other plants, suggesting that CBFs are evolutionarily conserved [10]. Nevertheless, a biochip expression analysis in tomato showed that genes regulated by CBFs in different plants respond in via different pathways [12]. Although CBFs play a crucial role in gene regulation during cold acclimation, according to bioinformatics analysis, they regulate and control only approximately 12% of genes in the COR transcriptome [13]. This finding indicates that most COR genes are governed by CBF-independent regulons.

*Rumex patientia* L. (2n = 10), a perennial herb belonging to the Polygonaceae family, is consumed as a green vegetable in several parts of the world, particularly in Turkey and India [14]. *R*. *patientia* can tolerate abiotic stress and withstand extremely low temperatures (-35°C) by relying on overwintering buds, which become dormant during severe winter months. This tolerance suggests that the species is suitable for cultivation in cold regions and for research on cold tolerance in plants [15,16]. The dried roots of *R*. *patientia* have also been employed for many decades as traditional medicines (e.g., as a purgative, antipyretic compound, depurative and tonic) for the treatment of wounds and diverse diseases [17]. The root extract of *R*. *patientia* contains anthraquinone, tannin, naphthalene and naphthoquinone derivatives [18]; it was first to North China introduced from Ukraine in 1995 as a foodstuff for animal husbandry [19]. Despite the abovementioned attributes, publicly available genetic databases contain limited reports regarding this species, and no nucleotide sequences from *R*. *patientia* have been deposited into the NCBI GenBank database to date.

Therefore, in this investigation, we used Illumina Hiseq technology for transcriptome assembly in *R*. *patientia* and compared transcriptomes under normal and cold conditions to evaluate how this species responds to cold stress. Our findings enhance our understanding of the *R*. *patientia* gene regulation response to cold stress and reveal novel approaches for improving cold tolerance through genetic engineering.

## Materials and methods

### Stress treatments and sample preparation

*R*. *patientia* seeds were grown in the greenhouse of Northeast Agricultural University (26°C, 16 h light photoperiod). After cultivation for two months, 20 seedlings were transferred to different treatment conditions. For this study, we set -5°C as a cold stress treatment because the temperature of soil is approximately -5°C in winter in Harbin, even though the air temperature is -30°C. Half of the plants were shifted to the cold stress conditions (-5°C for 4 h), and the other half were used as a control. The roots of treated plants were harvested after 4 h of treatment, frozen in liquid nitrogen and prepared for RNA extraction. Three completely independent harvesting experiments were repeated as biological replicates.

Total RNA was extracted from roots using the TRIzol according to the manufacturer’s protocol (TIANGEN, Beijing, China). The RNA purity and integrity were assessed using a Nanophotometer spectrophotometer (IMPLEN, CA, USA). Qualified RNA samples were used for cDNA synthesis with the PrimeScript™ RT Reagent Kit and gDNA Eraser (TaKaRa, Tokyo, Japan). cDNA fragments of 150–200 bp in length were selected for polymerase chain reaction (PCR) amplification, and a cDNA library was used for sequence analysis via Illumina sequencing at Novogene Research Pty. Ltd., Beijing, China.

### Transcriptome analysis under cold treatment

Raw data in fastq format were first processed through in-house Perl scripts. In this step, clean data were obtained by removing reads containing adapters, reads containing poly-N and low-quality reads from the raw data. All downstream analyses were based on high-quality clean data. The remaining clean reads were assembled into unigenes using the short-read assembly program SOAPdenovo [20]. All unigenes were then used in a blast search (E-value < 10^−5^) and annotated against various databases, including the NCBI non-redundant protein (Nr), NCBI nucleotide sequences (Nt), Kyoto Encyclopedia of Genes and Genomes Ortholog (KEGG), SwissProt, Protein family (PFAM), Gene Ontology (GO), and Cluster of Orthologous Groups (COG) databases.

### Identification and annotation of differentially expressed genes

Differential expression analysis between two treatments was performed using the DESeq R package (1.10.1). The false discovery rate (FDR) control method was applied in Benjamini and Hochberg method to correct the results for P-values. FDR < 0.01 and FC (fold change) ≥ 2 were set as the threshold to determine the significance of gene expression differences. For differentially expressed genes (DEGs), we considered an adjusted P-value < 0.05 identified by DESeq and a 2-fold or greater change in fragments per kilobase of gene per million mapped reads (FPKM). Regarding the functional annotation of DEGs, the results of GO enrichment analysis were evaluated using topGO R packages based on Wallenius non-central hyper-geometric distribution [21]. Finally, KOBAS software was employed to test the statistical enrichment of DEGs in KEGG pathways [22].

### Quantitative reverse transcription PCR (qRT-PCR) validation

Ten representative DEGs identified by RNA sequencing (RNA-Seq) were chosen for experimental validation by qRT-PCR using gene-specific primers (S1 Table). The reaction was performed using SYBR® Premix Ex Taq™ II Kit (Tli RNaseH Plus) (TaKaRa, Tokyo, Japan) in a volume of 20 μl containing 10 μl SYBR Premix Ex Taq (2×), 0.4 μl ROX Reference Dye II (10×), 2 μl cDNA template, and 0.5 μM each primer. Amplification was performed as follows: 95°C for 30 s followed by 45 cycles of 95°C for 5 s and 60°C for 40 s. All reactions were performed in biological triplicate, and the results are expressed relative to the expression levels of β-actin based on the 2^-ΔΔCt^ method [23].

## Results

### *De novo* assembly and functional annotation

After filtering adapters, reads containing poly-N and low-quality reads, ~ 2.7 million clean reads were purified from 2.9 million raw reads containing a total of 3.4 G nucleotides with a high percentage of clean reads (81.14%-98.16%) (Table 1). Six sample transcripts (approximately 20.69 Gbp of clean reads) were pooled together to perform *de novo* transcriptome assembly. Using the short-read assembly program SOAPdenovo, 161,869 transcripts were assembled with an N50 of 1412 bp. A total of 115,589 unigenes were produced from the assembled transcripts (Table 2).

**Table 1:** Sequencing output statistics of control and cold-treated *R*. *patientia*.

	Samples					
	Control_1	Control_2	Control_3	Treated_1	Treated_2	Treated_3
**Number of raw reads**	**30106792**	**26387332**	**28098657**	**35182884**	**26821572**	**30472196**
**Number of clean reads**	**29212660**	**25902104**	**27406323**	**34122498**	**24095683**	**24725848**
**Percentage of clean reads**	**97.03%**	**98.16%**	**97.54%**	**96.99%**	**89.84%**	**81.14%**
**Size of clean reads**	**3.65Gbp**	**3.24Gbp**	**3.43Gbp**	**4.27Gbp**	**3.01Gbp**	**3.09Gbp**
**Number of total clean reads**	**20.69Gbp**					

**Table 2:** Statistics of transcriptome assemble and unigenes.

	Number of transcripts	Number of unigenes
**Number**	**161,869**	**115,589**
**Total nucleotide**	**128,889,868**	**76,040,314**
**Minimum length**	**201**	**201**
**Median length**	**426**	**353**
**Maximum length**	**15363**	**15363**
**Mean length**	**796**	**658**
**N50**	**1412**	**1101**

**Fig 1:**
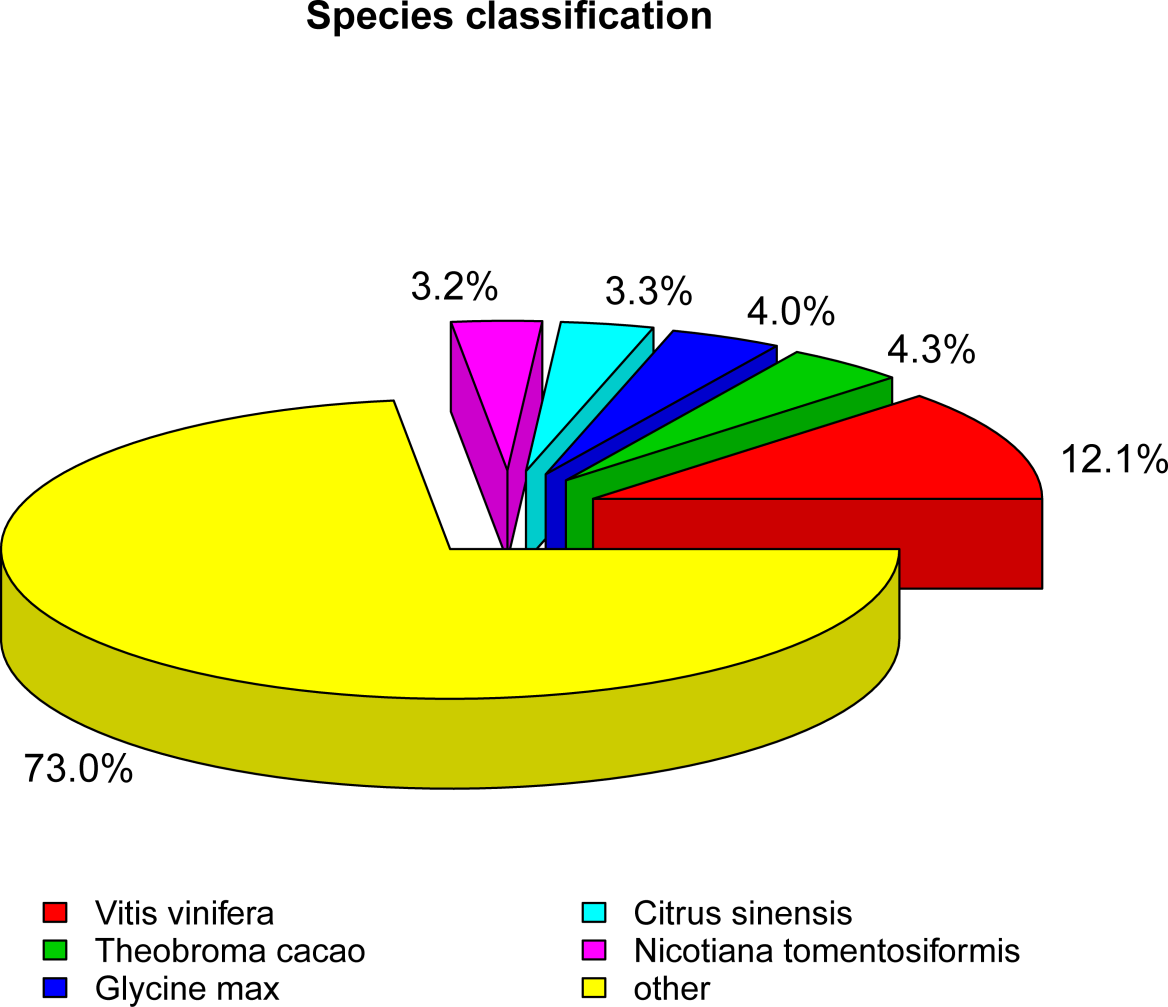
Homology search of *R*. *patientia* unigenes with other species by BLASTx to the NR database.

To identify the putative functions of unigenes in *R*. *patientia*, all of the assembled unigenes were functionally annotated against seven databases (Table 3 and S2 Table). Of 115,589 unigenes, 44,927 (38.86%), 28,271 (24.45%), 16,653 (14.4%), 35,947 (31.09%), 35,148 (30.4%), 35,784 (30.95%) and 19,129 (16.54%) were aligned to known sequences in the Nt, Nr, KEGG, SwissProt, PFAM, GO, and COG databases, respectively. Overall, 7,444 unigenes showed homology with known genes in all databases. Among these databases, the highest number of unigene annotations was in Nr, whereas the lowest number of annotations was matched in the KO database. As no genomic information on *R*. *patientia* has been published to date, we blast searched the unigenes against other species. The results showed the highest similarity to sequences from *Vitis vinifera* (5415); the next closest matches were *Theobroma cacao* (1952), *Glycine max* (1805), *Citrus sinensis* (1501) and *Nicotiana tomentosiformis* (1459), with comparable homology among these species (Fig 1 and S1 Fig).

**Table 3:** The annotation results of unigenes in seven databases.

	Number and percentage of Unigenes
**Annotated in Nr**	**44927 (38.86%)**
**Annotated in Nt**	**28271 (24.45%)**
**Annotated in KEGG**	**16653 (14.4%)**
**Annotated in SwissProt**	**35947 (31.09%)**
**Annotated in PFAM**	**35148 (30.4%)**
**Annotated in GO**	**35784 (30.95%)**
**Annotated in COG**	**19129 (16.54%)**
**Annotated in all databases**	**7444 (6.44%)**
**Annotated in at least one database**	**60157 (52.04%)**

**Fig 2:**
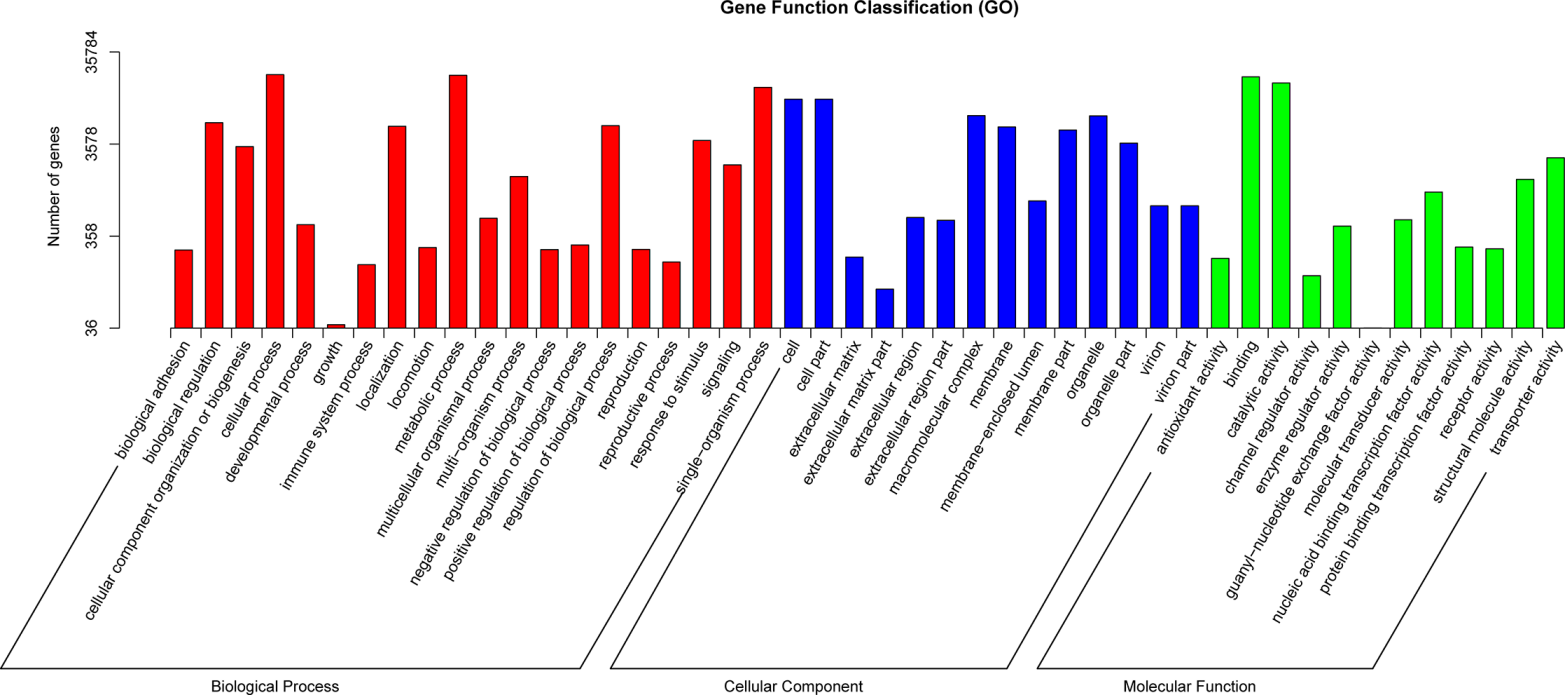
Functional annotation of unigenes based on Gene Ontology (GO) classification.

**Fig 3:**
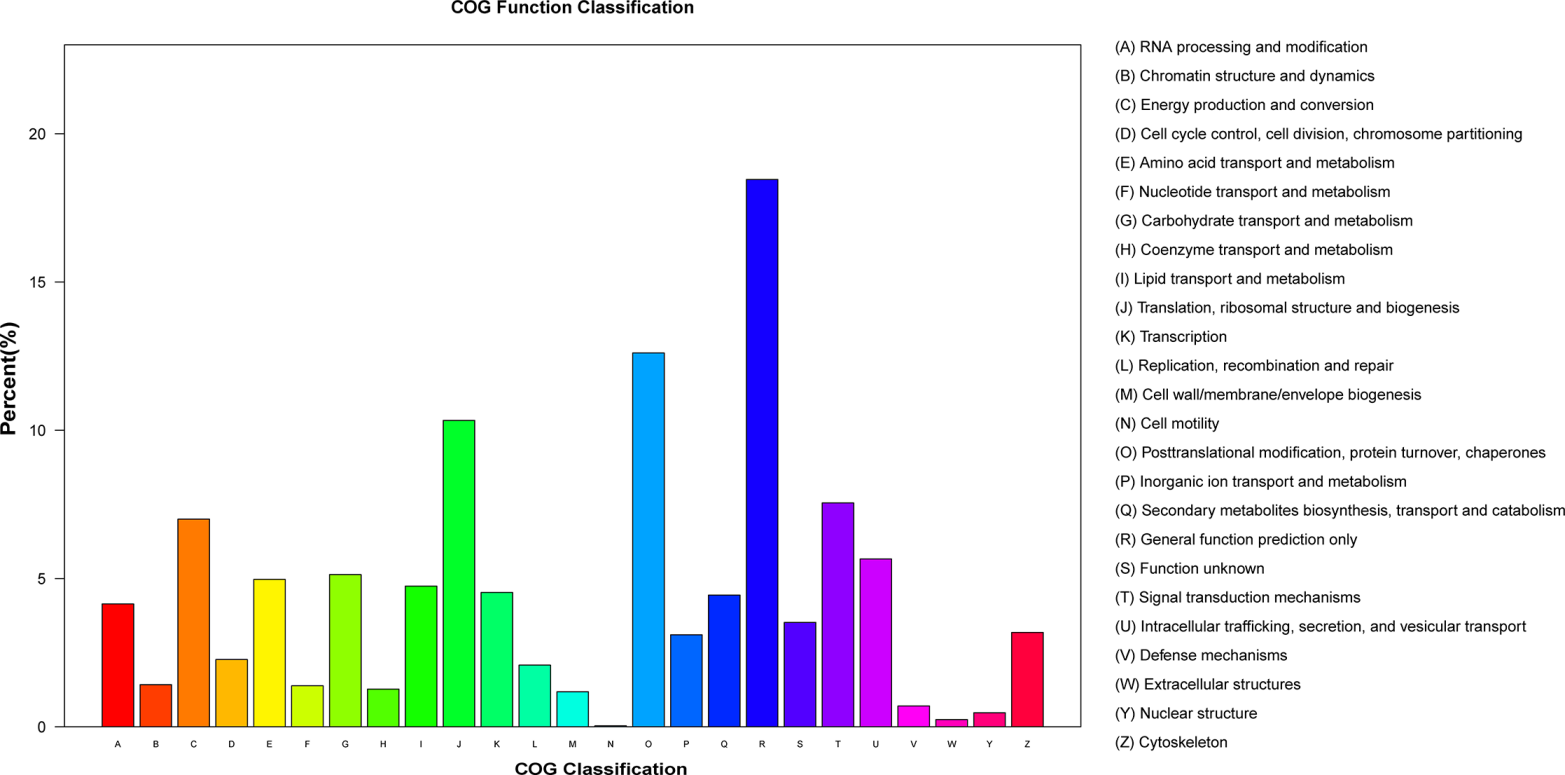
Distribution of genes in the transcriptome with COG functional classification. A total of 19129 sequences have a COG classification among 25 categories.

GO analysis fully described the genes and their biological functions in the classification system, which organized the functions of predicted unigenes into three main categories: biological processes, molecular function and cellular component (Fig 2). A total of 35,784 sequences were successfully assigned GO terms, among which 86,291 unigenes were assigned at least one biological process GO term, 54,651 to cellular components, and 42,798 to molecular functions. Furthermore, we performed phylogenetic classification using the COG database. A total of 19,129 unigenes were matched and clustered into 25 functional classes (Fig 3). The groups ‘General function prediction only’ (3,531) and ‘Transcription’ (2,412) were the two largest clusters, accounting for nearly 18.46% and 12.62% of the unigenes, respectively (Table 3 and Fig 3).

**Fig 4:**
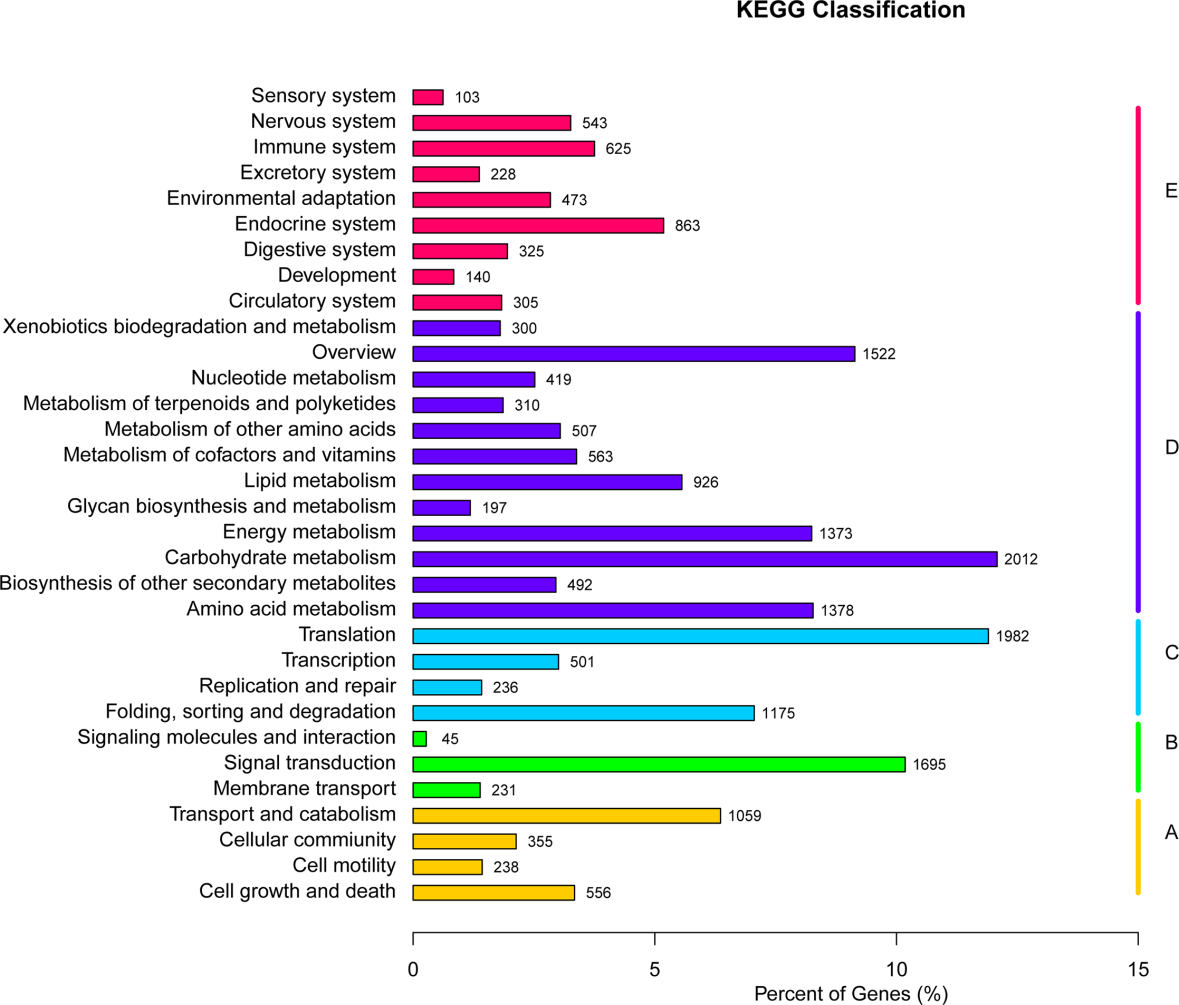
KEGG pathway classification of *R*. *patientia* unigenes.

Additionally, all of the assembled unigenes were further classified into KEGG functional subcategories. According to biological processes, a large number of genes involved in metabolic and cellular processes were highly represented, which comprised 20,008 and 20,294, respectively, of the matched unigenes in the subcategory, suggesting that cold temperature affects this cold-tolerant species by altering protein synthesis and metabolism (Fig 4). In the cellular component, the largest subcategory was cells (30.77%), and the second largest was cell parts (30.76%). Regarding molecular function, the largest numbers were found in catalytic activity (46.17%) and binding (53.8%). KEGG analysis was conducted to identify and predict active biochemical pathways in *R*. *patientia*, with 16,653 unigenes matching to 280 different KEGG pathways (S3 Table). Among these pathways, the most highly represented were carbon pathways (with 2,012 members), followed by translation (1,982) and signal transduction (1,695).

### Changes in gene expression in cold-treated *R*. *patientia*

**Fig 5:**
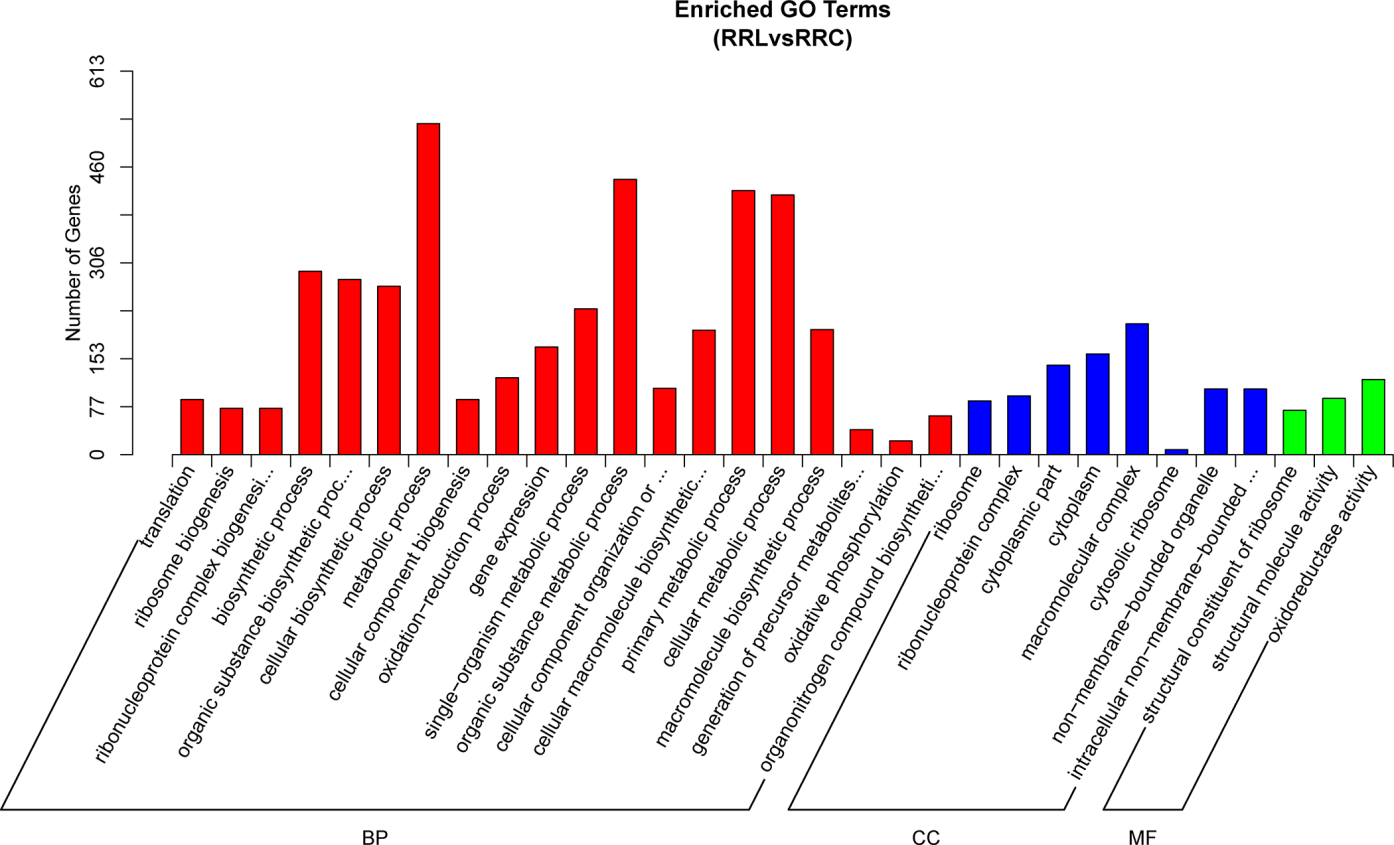
Functional annotation of differentially expressed genes based on Gene Ontology (GO) classification.

To better understand the genes that respond to cold stress in *R*. *patientia*, we identified putative DEGs in control and stress-treated samples using DESeq R package (padj < 0.05). In three control samples, 78,337 putative DEGs were detected. In contrast, 69,638 were found in cold-treated plants, of which 48,246 unigenes in common were detected in six libraries; 21,392 unigenes were identified in cold-treated samples, indicating that 30.72% of genes might be related to chilling stress (S2 Fig). In total, 1179 unigenes that were identified as DEGs, including up-regulated (925) and down-regulated genes (254), were successfully assigned GO annotations and classified into three major metabolic pathways (Fig 5, S2 Table and S3 Fig). Metabolic process (69.06%), macromolecular complex (27.28%) and oxidoreductase activity (15.67%) were the largest subcategories in biological processes, cellular component and molecular function, respectively. To help us better understand the biological function of DEGs under cold stress in *R*. *patientia*, KEGG pathway analysis was performed, annotating the DEGs into 10 top pathways (S3 Table and S4 Table). Ribosome, carbon metabolism, oxidative phosphorylation and biosynthesis of amino acids were the most highly enriched pathways according to KEGG analysis.

### Validation of *R*. *patientia* DEGs after cold stress

Table 4 lists the 10 most up- and down-regulated DEGs and their annotations after cold treatment. Among the top 10 up-regulated DEGs, four putative orthologs of the CRT/DRE-binding factor of *Arabidopsis* were identified, which is one of the CBF transcription factors induced by low, nonfreezing temperatures. Blast hits against known proteins were found for only two down-regulated DEGs; another eight were annotated as unknown functions, and these may constitute novel factors induced by cold stress, which requires further study.

**Table 4:** The 10 most up- and down-regulated *R*. *patientia* genes and annotations after cold stress.

Gene ID	Log2(fold-change)	Description	Type
**c68449_g1**	**8.5563**	**CRT/DRE binding factor**	**up**
**c63591_g1**	**8.2511**	**CRT/DRE binding factor**	**up**
**c76039_g1**	**7.8142**	**zinc finger protein CONSTANS-LIKE 1**	**up**
**c85484_g1**	**7.2464**	**NA**	**up**
**c76748_g3**	**7.0709**	**CRT/DRE binding factor**	**up**
**c72584_g1**	**6.8482**	**Aldo/Keto Reductase**	**up**
**c74692_g1**	**6.6489**	**unsaturated rhamnogalacturonyl hydrolase YesR-like**	**up**
**c76748_g1**	**6.5762**	**CRT/DRE binding factor**	**up**
**c63095_g1**	**6.5039**	**NA**	**up**
**c76501_g1**	**6.4841**	**hypothetical protein COCSUDRAFT_21535**	**up**
**c50023_g1**	**-7.3423**	**LINE-1 retrotransposable element ORF2 protein**	**down**
**c68197_g6**	**-7.0881**	**NA**	**down**
**c57726_g1**	**-6.9272**	**NA**	**down**
**c57779_g2**	**-6.053**	**NA**	**down**
**c38504_g1**	**-5.7153**	**NA**	**down**
**c68060_g2**	**-5.1872**	**putative non-LTR retroelement reverse transcriptase**	**down**
**c78553_g1**	**-4.8282**	**NA**	**down**
**c58200_g1**	**-4.5248**	**NA**	**down**
**c66306_g1**	**-4.3839**	**NA**	**down**
**c81134_g2**	**-4.3242**	**NA**	**down**

**Fig 6:**
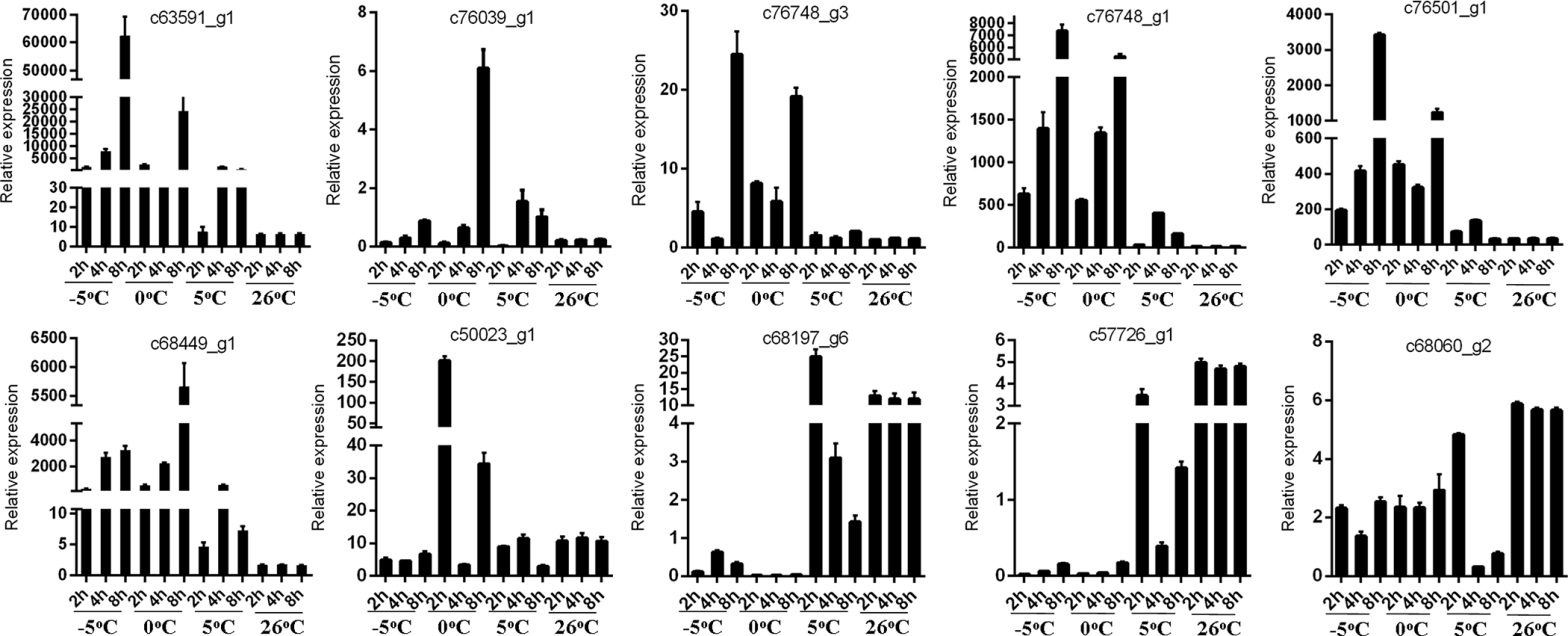
The expression of *R*. *patientia* genes in response to chilling at different temperature for 0 to 8 h as determined by qRT-PCR.

In total, we tested and validated 10 of the top 20 DEGs by qRT-PCR to examine their expression after cold stress for 2, 4, and 8 h. Expression of all 6 up-regulated genes was enhanced by chilling, although their expression changed with the length of chilling treatment and the specific temperature (Fig 6). Consistent with the RNA-Seq results, all four of the most down-regulated genes were repressed by chilling (Fig 6). The consistency between the results of qRT-PCR and the RNA-Seq analyses confirmed the validity of the *de novo*-assembled transcriptome and our evaluation of the cold stress regulation of the transcriptome.

### Analysis of *R*. *patientia* unigenes involved in the cold response pathway

The CBF transcriptional cascade, a crucial pathway for regulating gene expression under low temperatures, is conserved in diverse plant species. Using our RNA-Seq data, we identified CBF pathway genes to more deeply understand the genes involved in the response to low temperature. Overall, 66 up-regulated genes were identified as putatively involved in the response to cold stress, and their corresponding CBF pathway orthologs are listed in S5 Table. As shown in S5 Table, 25 unigenes were matched with zinc finger family (Znf) members, representing the largest group in *R*. *patientia*. The second largest group was COR (15 unigenes), including a large proportion of Ca^2+^-binding transcription factors involved in the response to cold stimuli. Genes in the AP2/ERF family encode transcriptional regulators with a variety of functions involved in the developmental and physiological processes in plants. In our transcriptome, two subfamilies of AP2/ERF transcriptome factors were identified: DREBs (c76748_g3, c63591_g1, c68449_g1, c53582_g1, and c76748_g4) and ethylene-responsive transcription factors (c74787_g1, c67493_g1, c63394_g1, c32287_g1 and c54408_g1).

Three unigenes (c80957_g2, c81107_g2 and c65606_g1) were found to harbor a domain similar to the MYB-like DNA-binding domain, which can alter transcription when plants are exposed to low temperatures. Three unigenes (c74749_g1, c63109_g2 and c63109_g3) contain the NAC domain, which responds to biotic and abiotic stresses. In addition, 4 unigenes (c71662_g1, c89475_g1, c72305_g1, and c72369_g4) show high similarity to the bZIP transcription factor sequence, which is involved in regulating expression of a subset of COR genes.

## Discussion

*R*. *patientia* L. is a new type of high-protein forage feed and is also a good ground cover plant for effectively preventing soil erosion and improving the ecological environment [24,25]. The tolerance of *R*. *patientia* to extremely low temperatures is attracting research attention, as this species may be suitable for growth in cold regions. In this investigation, we analyzed the transcriptome sequences of *R*. *patientia* under cold acclimation and obtained 20.69 Gbp of clean reads from six samples (Table 1). Compared with the control sample, 925 up-regulated genes and 254 down-regulated genes were identified as DEGs, indicating that the altered genes are involved in regulating the response to cold treatment. Similar to previous reports from many other species, the number of DEGs identified under cold stress was increased compared with the control [26–28]. Interestingly, among the 925 up-regulated transcripts, 91 (9.8%) had unknown/unclassified functions (S2 Table). This result suggests that the presence of putative novel genes might be specific to *R*. *patientia*, and these genes may be involved in important pathways in cold stress adaptation.

According to functional annotations based on the GO and KEGG databases, the DEGs identified by cold stress are mainly involved in the ribosome, carbon metabolism and oxidative phosphorylation pathways (S4 Table). The largest number of DEGs was related to the ribosome pathway (ko03010), suggesting that transcription and translation are vigorous in *R*. *patientia* after cold stress and that there is an additional gradual increase in gene participation during a prolonged stress-response period. Similar findings have been reported in the transcriptomes of many other plants under low temperature [29–31]. Carbon metabolism (ko01200) was the second largest group related to the identified DEGs, suggesting that low, nonfreezing temperatures may increase the concentration of carbon compounds and their primary biosynthetic enzymes. Soluble sugars not only function as a source of metabolic energy but also act as molecular signals regulating different genes associated with stress pathways to cope with abiotic stress conditions [32]. Sucrose was previously shown to be the most abundant soluble sugar in *Spinacia oleracea* during cold stress via a mechanism that involves increasing sucrose phosphate synthase activity [33]. In the process of cold tolerance in plants, activated starch enzymes hydrolyze starch into sugars, which increases water retention and osmotic potential in plant cells [34].

The oxidative phosphorylation pathway (ko00190) includes enzymes that oxidize nutrients, releasing energy as ATP and producing most of the energy in mitochondria [35]. This pathway is most likely pervasive during a plant’s lifespan. A total of 34 up-regulated DEGs were successfully annotated to this pathway, representing the third largest cluster, and their high levels demonstrated that the energy released by oxidation mainly leads to the reformation of ATP, which may serve as an emergency resistance measure related to a low-temperature environment. This result was consistent with findings for the *Anthurium* transcriptome, whereby significant enrichment was identified in similar top pathways after cold treatment. However, the opposite results were obtained in chilled corn shoots, in which the efficiency of oxidative phosphorylation was markedly reduced. One possible reason for this low level of efficiency in corn might be that the energy released was consumed to adapt to a cold environment [36]. In addition, accumulation of proline was artificially increased under cold stress, which might act as a signal for reconfiguring gene expression [10]. At low temperatures, ammonium absorption is enhanced by the elevation of glutamine synthetase activity in rice roots, indicating that low temperatures can modulate nitrogen metabolism [37]. One striking finding in our study was the identification of 30 DEGs involved in the biosynthesis of amino acids, indicating that *R*. *patientia* can protect itself against cold stress.

During the process of cold adaptation, expression of a large number of COR transcription factor genes is activated by receptor proteins or signal transduction pathways to enhance oxidation resistance and osmotic adjustment ability and to promote reconstruction and material balance in the cell membrane system. In *R*. *patientia*, 66 up-regulated genes were annotated as putative CBF transcription factors, including members of the MYB, AP2/ERF, CBF, Znf, bZIP, NAC and COR families, which is consistent with the cold resistance and tolerance of this species (S5 Table). The AP2/ERF family of transcription factors, among the largest groups in *R*. *patientia*, contains four major subfamilies: the AP2, ERF, RAV and DREB subfamilies [38,39]. The DREB subfamily consists of a well-known set of transcription factors that have been detected and cloned in numerous species and that are known to activate expression of abiotic stress-responsive genes via specific binding to a DRE/CRT *cis*-acting element [40,41]. In this investigation, five DREB genes were successfully matched and showed significant increases after cold stress, suggesting that the DREB1/CBF pathway functionally acts as a key transcription factor in the regulation of the cold response in *R*. *patientia*.

Zinc-finger transcription factors play a central role in regulating various signal transduction pathways in plants [42,43]. Previous studies have suggested that the overexpression of CBFs that control the transcription of numerous downstream stress-related genes can enhance Zat10 and Zat12 expression in *Arabidopsis*, and CBF-induced increases in the expression of a set of COR genes were positively correlated with Zat10 and Zat12 in response to cold [10,44]. The large number of up-regulated genes annotated as Znfs in *R*. *patientia* indicates that these transcription factors might participate in the regulating the response to cold. Zhang et al. [45] demonstrated that several newly discovered bZIP genes in wheat were strongly induced by multiple abiotic stresses through the abscisic acid (ABA) signaling pathway. However, in the present work, we were unable to verify which signaling pathway might be associated with bZIP in *R*. *patientia* based only on the obtained transcriptome sequences. Nonetheless our observations demonstrate that bZIP expression is advantageous for the response to cold conditions.

The COR transcription factor family comprises Ca^2+^-binding proteins [8]. In this study, the second largest family of cold-stress transcription factors identified in *R*. *patientia* was Ca^2+^-binding proteins, suggesting that these proteins are jointly involved in the response to cold stress. There have been similar discoveries in *Medicago sativa* cells and *Brassica napus* leaves, in which cold stress-induced plasma membrane rigidification resulted in actin cytoskeletal rearrangement, induction of Ca^2+^ channels, and an artificial increase in cytosolic Ca^2+^ levels. These changes further induce expression of COR genes and cold acclimation in plants [5,46]. Indeed, a transient increase in the cellular Ca^2+^ concentration in response to dehydration and low temperature appear to stimulate cellular signaling processes [38].

## Conclusion

To our knowledge, this investigation is the first to provide a COR transcriptome assembly in *R*. *patientia*, a species with little or no available genomic data, which has limited its economic utility for cultivation in cold regions. After an in-depth RNA-Seq analysis, we obtained and annotated 60,157 assembled unigenes to at least one database. A large number of potential COR genes were identified, suggesting that this species is suitable for cultivation in northern China. Our findings of up-regulated DEGs suggested that cold stimuli greatly affect protein translation and cellular metabolism in this species. A large number of the identified unigenes in the CBF pathway and COR genes were induced by low temperatures, which was consistent with the strong cold tolerance of this species. In summary, these data provide valuable information for future research and genomic studies in *R*. *patientia*.

## Supporting information

S1 Table DNA oligos used in qRT-PCR. (XLSX)

S2 Table All the assembled unigenes functionally annotated against seven databases. (XLS)

S3 Table All unigenes matched to different KEGG pathways. (XLS)

S4 Table The top 10 of KEGG pathways of differentially expressed genes of *R*. *patientia*. (DOC)

S5 Table Up-regulated unigenes matched to known cold-stress transcription factors in *R*. *patientia*. (DOC)

S1 Fig Unweighted pair group method with arithmetic mean dendrogram of different species based on Jaccard’s coefficient of shared unigenes. (DOC)

S2 Fig Cluster analysis of expression level (A) and Venn diagram (B) of putative DEGs in *R*. *patientia* (fold changes > 2, false discovery rate < 0.01).
(DOC)

S3 Fig Volcanoplot analysis of DEGs in *R*. *patientia* (the scattered point in diagram is DEG, blue dot represents no significantly different gene, red dot shows differentially expressed gene). (DOC)
