# Correction: *De novo* assembly and analysis of the transcriptome of *Rumex patientia* L. during cold stress

**DOI:** 10.1371/journal.pone.0190154

**Published:** 2017-12-19

**Authors:** Jianxin Liu, Yongqing Xu, Liguo Zhang, Wei Li, Zhenxue Cai, Fei Li, Mu Peng, Fenglan Li, Baozhong Hu

The affiliations for authors Liguo Zhang and Wei Li are incorrect. Liguo Zhang and Wei Ling are not affiliated with #1 but with #2 Heilongjiang Academy of Agricultural Sciences, Harbin, China.

## References

[1] Liu J, Xu Y, Zhang L, Li W, Cai Z, Li F, et al (2017) *De novo* assembly and analysis of the transcriptome of *Rumex patientia* L. during cold stress. PLoS ONE 12(10): e0186470 https://doi.org/10.1371/journal.pone.0186470 29023590 10.1371/journal.pone.0186470 PMC5638559

