# Supplementary material for: De novo assembly and analysis of the transcriptome of Rumex patientia L. during cold stress
Source: PLoS One. 2017 Oct 12;12(10):e0186470. doi: 10.1371/journal.pone.0186470 (PMC5638559; doi:10.1371/journal.pone.0186470)
Supplement: S4 Table — (DOC) [file pone.0186470.s004.doc]

S4 Table The top 10 of KEGG pathways of differentially expressed genes of *R. patientia*

| Pathway ID | Pathway term | *q*-value | Gene number |
| --- | --- | --- | --- |
| ko03010 | Ribosome | 4.03E-09 | 85 |
| ko01200 | Carbon metabolism | 0.193445 | 43 |
| ko00190 | Oxidative phosphorylation | 0.001981 | 34 |
| ko01230 | Biosynthesis of amino acids | 0.184298 | 30 |
| ko00020 | Citrate cycle (TCA cycle) | 0.000868 | 24 |
| ko00010 | Glycolysis / Gluconeogenesis | 0.441181 | 24 |
| ko03013 | RNA transport | 0.515526 | 19 |
| ko00620 | Pyruvate metabolism | 0.743086 | 17 |
| ko00500 | Starch and sucrose metabolism | 0.251602 | 16 |
| ko04141 | Protein processing in endoplasmic reticulum | 0.741479 | 14 |
