# Supplementary material for: De novo assembly and analysis of the transcriptome of Rumex patientia L. during cold stress
Source: PLoS One. 2017 Oct 12;12(10):e0186470. doi: 10.1371/journal.pone.0186470 (PMC5638559; doi:10.1371/journal.pone.0186470)
Supplement: S5 Table — (DOC) [file pone.0186470.s005.doc]

S5 Table Up-regulated unigenes matched to known cold-stress transcription factors in *R. patientia*

| Unigenes ID | Annotation | Ortholog | Log2 ratio | *p*-value |
| --- | --- | --- | --- | --- |
| c80957_g2 | MYB-like DNA-binding domain | MYB | 2.2116 | 5.50E-05 |
| c81107_g2 | MYB-related transcription factor LHY | MYB | 1.679 | 8.85E-05 |
| c65606_g1 | MYB-like DNA-binding domain | MYB | 1.1054 | 0.000246 |
| c76748_g3 | Dehydration-responsive element-binding protein | AP2/ERF | 7.0709 | 2.06E-17 |
| c63591_g1 | Dehydration-responsive element-binding protein | AP2/ERF | 8.2511 | 1.99E-16 |
| c68449_g1 | Dehydration-responsive element-binding protein | AP2/ERF | 8.5563 | 2.74E-16 |
| c53582_g1 | Dehydration-responsive element-binding protein | AP2/ERF | 3.85 | 6.50E-08 |
| c76748_g4 | Dehydration-responsive element-binding protein | AP2/ERF | 5.7844 | 3.08E-07 |
| c74787_g1 | Ethylene-responsive | AP2/ERF | 2.6039 | 1.72E-10 |
| c67493_g1 | Ethylene-responsive | AP2/ERF | 2.8684 | 8.33E-10 |
| c63394_g1 | Ethylene-responsive | AP2/ERF | 2.4996 | 2.21E-07 |
| c32287_g1 | Ethylene-responsive | AP2/ERF | 3.6602 | 7.94E-06 |
| c54408_g1 | Ethylene-responsive | AP2/ERF | 4.0869 | 0.000476 |
| c53582_g1 | C-repeat binding factor | CBF | 3.85 | 6.50E-08 |
| c76748_g4 | C-repeat binding factor | CBF | 5.7844 | 3.08E-07 |
| c68048_g1 | Histone-like transcription factor (CBF/NF-Y) | CBF | 2.2796 | 4.16E-06 |
| c72388_g2 | CCAAT-binding transcription factor (CBF-B/NF-YA) | CBF | 1.4678 | 1.18E-05 |
| c68287_g4 | Histone-like transcription factor (CBF/NF-Y) | CBF | 2.2737 | 1.44E-05 |
| c65551_g1 | Histone-like transcription factor (CBF/NF-Y) | CBF | 1.9746 | 1.77E-05 |
| c76039_g1 | Zinc finger | Znf | 7.8142 | 3.96E-36 |
| c53869_g1 | Zinc finger | Znf | 3.4118 | 6.00E-12 |
| c62027_g1 | Zinc finger | Znf | 2.2362 | 2.04E-09 |
| c71565_g1 | RING-H2 zinc finger | Znf (RING-Finger) | 1.8186 | 1.10E-08 |
| c76431_g1 | Zinc finger | Znf | 2.766 | 2.10E-07 |
| c75942_g1 | RING-H2 zinc finger | Znf (RING-Finger) | 1.8979 | 4.97E-07 |
| c47191_g1 | Zinc finger | Znf | 4.9907 | 1.67E-06 |
| c121314_g1 | Zinc finger | Znf | 3.6984 | 4.71E-06 |
| c67118_g1 | Zinc finger | Znf | 2.6344 | 6.09E-06 |
| c80254_g1 | Zinc finger | Znf | 1.7592 | 1.58E-05 |
| c75460_g2 | Zinc finger | Znf | 2.1522 | 3.59E-05 |
| c13978_g1 | Zinc finger | Znf | -- | 6.29E-05 |
| c75947_g1 | Zinc finger | Znf | 2.8094 | 6.42E-05 |
| c53959_g1 | Zinc finger | Znf | 3.033 | 7.39E-05 |
| c70862_g1 | Zinc finger | Znf | 3.3136 | 8.65E-05 |
| c65620_g1 | Zinc finger | Znf | 1.0916 | 0.000187 |
| c67509_g1 | Zinc finger | Znf | 2.4437 | 0.000194 |
| c69775_g1 | Zinc finger | Znf | 4.9185 | 0.000269 |
| c56881_g1 | Zinc finger | Znf | 5.9489 | 0.000283 |
| c67787_g1 | Zinc finger | Znf | 1.1988 | 0.000304 |
| c68591_g2 | zinc finger | Znf | -- | 0.000327 |
| c52774_g1 | Zinc finger | Znf | 1.5864 | 0.000338 |
| c62989_g1 | WRKY | Znf (WRKY) | 2.743 | 1.49E-16 |
| c60635_g1 | WRKY | Znf (WRKY) | 1.1022 | 0.000165 |
| c75986_g1 | WRKY | Znf (WRKY) | 1.0466 | 0.00047 |
| c74749_g1 | NAC domain | NAC | 2.4805 | 1.63E-09 |
| c63109_g2 | NAC domain | NAC | 3.8209 | 2.04E-09 |
| c63109_g3 | NAC domain | NAC | 3.72 | 6.54E-06 |
| c71662_g1 | bZIP transcription factor | bZIP | 5.0763 | 1.26E-06 |
| c89475_g1 | bZIP transcription factor | bZIP | 1.0404 | 0.000165 |
| c72305_g1 | bZIP transcription factor | bZIP | 1.9511 | 0.000177 |
| c72369_g4 | bZIP transcription factor | bZIP | 2.0305 | 0.000204 |
| c64882_g1 | Calcium-transporting ATPase | COR | 4.8165 | 5.21E-20 |
| c67329_g1 | Calcium ion binding | COR | 3.2712 | 2.00E-14 |
| c66637_g3 | Calcium ion binding | COR | 3.0965 | 3.58E-11 |
| c74257_g1 | Calcium ion binding | COR | 3.0101 | 3.20E-08 |
| c76453_g1 | Calcium-dependent protein | COR | 2.578 | 9.04E-08 |
| c72740_g1 | Calcium ion transporter | COR | 3.7721 | 8.41E-07 |
| c73158_g1 | Calcium ion binding | COR | 2.8291 | 1.75E-06 |
| c74229_g1 | Calcium-binding | COR | 1.9715 | 4.85E-06 |
| c57929_g1 | Calcium ion transporter | COR | 3.2858 | 6.16E-06 |
| c61806_g1 | Calcium ion binding | COR | 2.5037 | 9.62E-06 |
| c71099_g1 | Calcium ion binding | COR | 1.1775 | 3.71E-05 |
| c74600_g1 | Calcium ion binding | COR | 1.6812 | 7.94E-05 |
| c69392_g1 | Calcium-binding | COR | 2.4461 | 0.00017 |
| c80918_g2 | Calcium ion binding | COR | 1.4243 | 0.000333 |
| c50181_g1 | Calcium ion transporter | COR | 5.2431 | 0.000407 |
