# Supplementary material for: De novo assembly and analysis of the transcriptome of Rumex patientia L. during cold stress
Source: PLoS One. 2017 Oct 12;12(10):e0186470. doi: 10.1371/journal.pone.0186470 (PMC5638559; doi:10.1371/journal.pone.0186470)
Supplement: S1 Fig — (DOC) [file pone.0186470.s006.doc]

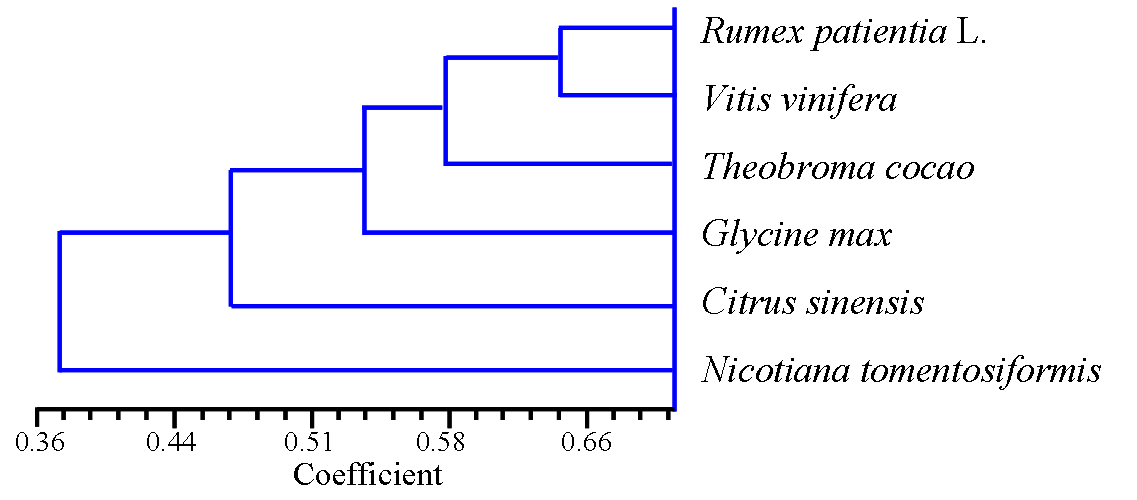


S1 Fig Unweighted Pair Group Method with Arithmetic Mean dendrogram of different species based on Jaccard’s coefficient of shared unigenes
