# Supplementary material for: De novo assembly and analysis of the transcriptome of Rumex patientia L. during cold stress
Source: PLoS One. 2017 Oct 12;12(10):e0186470. doi: 10.1371/journal.pone.0186470 (PMC5638559; doi:10.1371/journal.pone.0186470)
Supplement: S2 Fig — Cluster analysis of expression level (A) and Venn diagram (B) of putative DEGs in R. patientia (fold changes > 2, false discovery rate < 0.01). (DOC) [file pone.0186470.s007.doc]

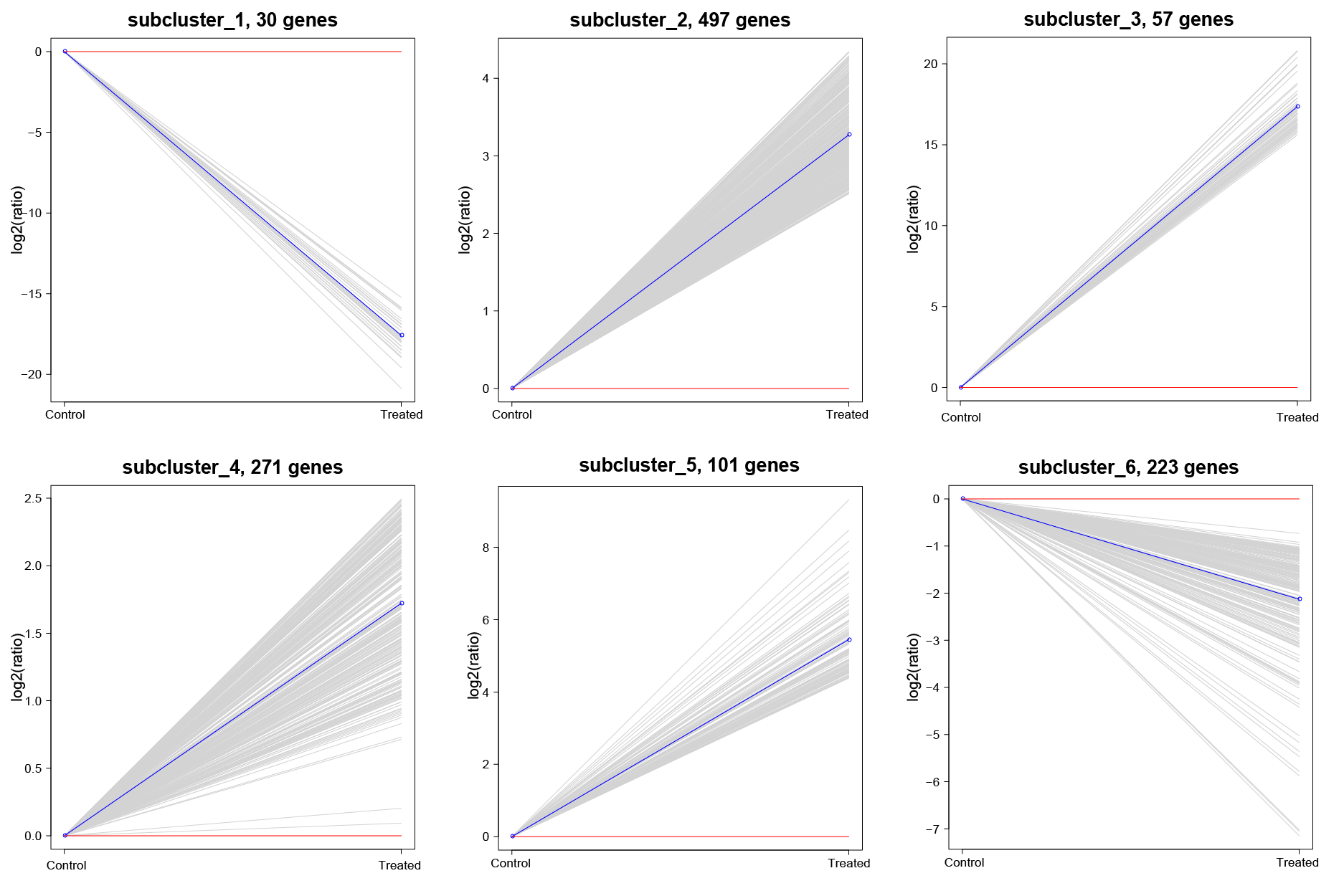

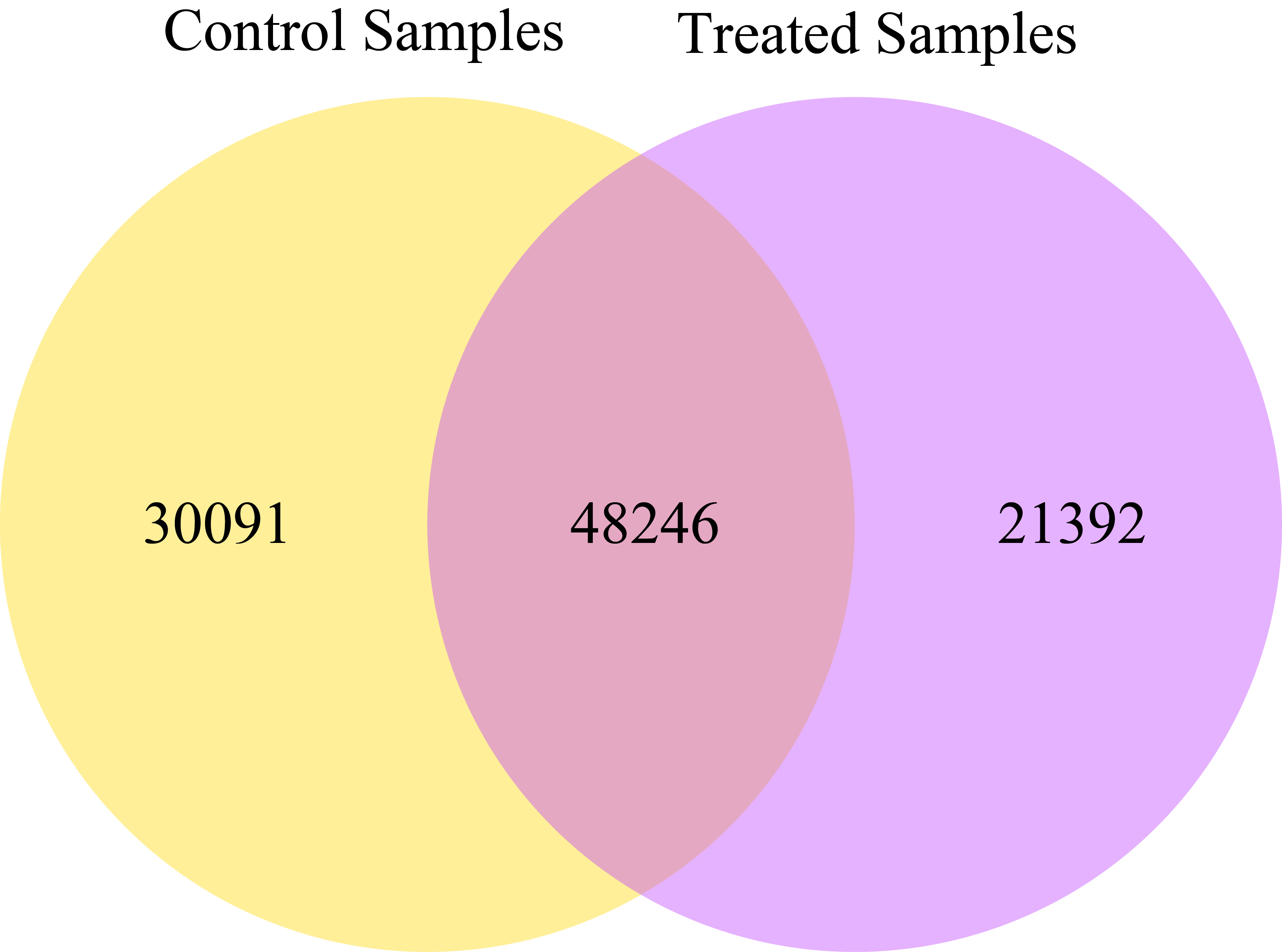


S2 Fig. Cluster analysis of expression level (A) and Venn diagram (B) of putative DEGs in *R. patientia* (fold changes > 2, false discovery rate < 0.01)
