# Supplementary material for: De novo assembly and analysis of the transcriptome of Rumex patientia L. during cold stress
Source: PLoS One. 2017 Oct 12;12(10):e0186470. doi: 10.1371/journal.pone.0186470 (PMC5638559; doi:10.1371/journal.pone.0186470)
Supplement: S3 Fig — (DOC) [file pone.0186470.s008.doc]

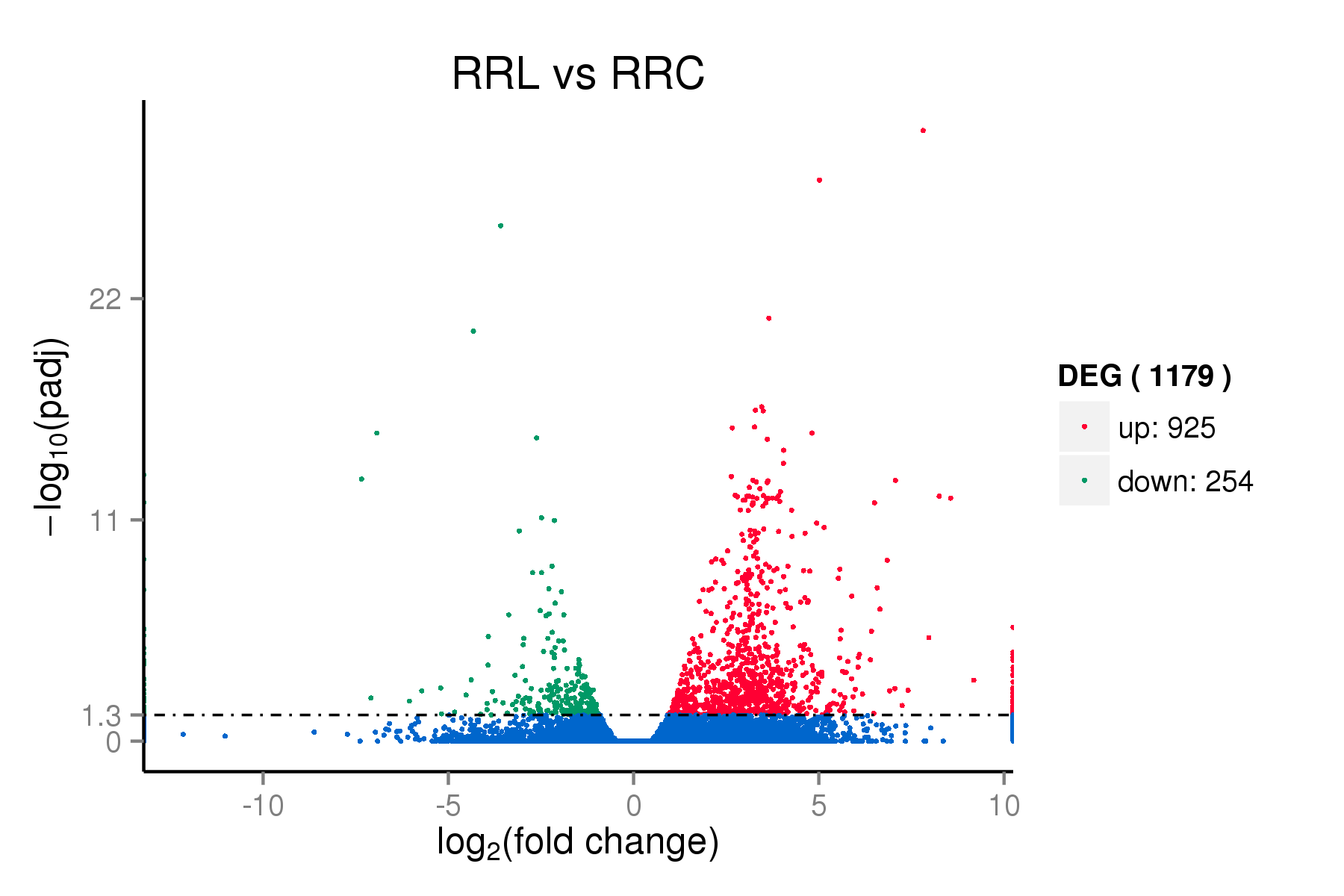


S3 Fig. Volcanoplot analysis of DEGs in *R. patientia* (the scattered point in diagram is DEG, blue dot represents no significantly different gene, red dot shows differentially expressed gene)
